# Whole grain diet reduces systemic inflammation: A meta-analysis of 9 randomized trials

**DOI:** 10.1097/MD.0000000000012995

**Published:** 2018-10-26

**Authors:** Yujie Xu, Qianyi Wan, Jinhua Feng, Liang Du, Ka Li, Yong Zhou

**Affiliations:** a West China School of Nursing and Department of Nursing; b Department of Gastrointestinal Surgery; c Department of Biliary Surgery, West China Hospital, Sichuan University; d Chinese Evidence-Based Medicine/Cochrane Center; e Department of nursing, West China Hospital, Sichuan University, Chengdu, China.

**Keywords:** C-reactive protein, inflammation, interleukin-6, meta-analysis, tumor necrosis factor-α, whole grain

## Abstract

**Backgrounds:**

Observational studies had suggested an inverse association between whole grain consumption and concentration of inflammatory markers, but evidence from interventional studies was inconsistent. Therefore, we conducted a meta-analysis of randomized trials to have a better understanding of this issue.

**Methods:**

This study has been registered in PROSPERO (ID: CRD42018096533). We searched PubMed, Web of Science, Embase, Medline, and Cochrane Library for articles focusing on the topic from inception to 1 January, 2018. Summary standardized mean difference (SMD) and 95% confidence interval (CI) were calculated by using either random effect model or fixed effect model according to the heterogeneity of included studies. Subgroup analysis was also performed.

**Results:**

Totally 9 randomized trials included 838 participants were identified. In a pooled analysis of all studies, consumption of whole grains had an inverse association with inflammatory markers (SMD 0.16, 95% CI, 0.02–0.30), including C-reactive protein (CRP), Interleukin-6 (IL-6), Tumor necrosis factor-α (TNF-α), Interleukin-1β (IL-1β). Specific analyses for CRP and IL-6 yielded that whole grain diet was related with a significant decrease in the concentration of CRP (SMD 0.29, 95% CI, 0.08–0.50) and IL-6 (SMD 0.19, 95% CI, 0.03–0.36).

**Conclusions:**

The evidence suggested that citizens could benefit from increased whole grain intake for reducing systemic inflammation. Further well-designed studies are required to investigate the mechanism under the appearance.

## 1 Introduction

Previous evidence indicated that inflammation was associated with cardiovascular diseases,^[1,2]^ certain cancer,^[3]^ and played a central role in the pathogenesis of metabolic diseases, including obesity, metabolic syndrome, and type 2 diabetes.^[4–7]^ Possible mechanisms in this relation included facilitation of atherosclerosis,^[8]^ development of insulin resistance,^[9]^ endoplasmic reticular stress,^[10]^ and change in gut microbiota.^[11]^ Several dietary factors had been reported to have an effect on the inflammatory markers. For instance, it had been reported that intake of red meat elevated inflammation, while consumption of fish,^[12]^ dairy,^[13]^ as well as nutrients such as zinc,^[14]^ and iron had an inverse association with the inflammatory markers.^[15]^ Moreover, Mediterranean-style diet which rich in whole grains, fruits, vegetables, legumes, and olive oil had been confirmed effective in reducing inflammation and its associated cardiovascular risk.^[16]^

Whole-grain foods had been reported to have a positive impact on reduced risk of developing type 2 diabetes,^[17,18]^ certain type of cancers,^[19–21]^ and cardiovascular diseases.^[22,23]^ This evidence led to health claims for increased consumption of whole-grain products in the USA, and Europe.^[24,25]^ However, the mechanisms by which whole grains exert their beneficial effects were not fully elucidated, a great deal of research had directly gone into the association between whole grain intake and inflammation. Among those exploration, observational studies had suggested a relation between whole grains and decreased concentrations of circulating inflammatory markers,^[26–28]^ possibly thanks to its bioactive compounds, such as vitamins B, E, zinc, fiber, and selenium.^[29]^ Nevertheless, various factors, including smoking status, alcohol use, physical activity, total calories, and other dietary compounds could confound these results, and it was difficult to adjust for them, thus intervention clinical trials which reduced confounding factors were warranted. But unfortunately, so far, the number of intervention studies was relatively small when compared to observations and showed inconsistent conclusion on this issue. A review published in 2013 had been concluded that available evidence could not indicate a clear relation between whole grains and inflammation by summarizing findings from earlier intervention studies.^[30]^ Another systematic review in the same year included 5 intervention on the topic up to 2010 and qualitatively described the association between whole grain and the concentration of inflammatory markers was not significant.^[31]^ While recently, Roager et al and Vanegas et al conducted a randomized cross-over trial and a randomized controlled study separately and drew into a consistent conclusion that whole grains did have an inverse association with a decreased concentration of inflammatory markers.^[32,33]^

It had been confirmed that whole grain intake could reduce the concentration of inflammatory markers in observational studies, but limited intervention trials did not reach consensus. However, to the best of our knowledge, no meta-analysis of intervention studies had been conducted. This meta-analysis aims to investigate the role of whole grain intake and the inflammatory markers with high-quality randomized trials.

## 2 Methods

This study has been registered in PROSPERO(ID: CRD42018096533). However, all analyses were based on previous published studies, thus no ethical approval and patient consent are required.

### 2.1 Search strategy and selection criteria

We did a systematic literature search of the PubMed, Web of Science, Embase, Medline, and Cochrane Library in order to identify randomized trials which reported the association between whole grain consumption and the inflammatory markers published before January 1, 2018. The following medical subject heading terms and text words were used in combination: “whole grain” or “cereal” or “wheat” or “brown rice”, “inflammat” or “C-reactive protein” or “CRP” or “interleukin-6” or “IL-6” or “tumor necrosis factor-α” or “TNF-α”, and “randomized trials”. No restrictions on language. We also carefully reviewed the reference lists of retrieved articles and published systematic reviews and meta-analyses to identify additional studies that could contribute to the general discussion on this topic.

To select eligible studies, several inclusion criteria according to the so-called PICOs checklists were used:

1) Population: people without taking any medicine that can change body inflammatory markers;
2) Intervention: diet that is rich in whole grain;
3) Control: refined grain diet adjusted for total calories intake;
4) Outcomes: the concentration of inflammatory markers;
5) Study design: randomized controlled studies or randomized cross-over trials. Case report, editorial letters, science reviews, and expert opinions were excluded.

### 2.2 Data extraction and quality assessment

Two researchers (Yujie Xu and Qianyi Wan) independently performed data extraction from the retrieved studies. A third researcher (Ka Li) resolved any discrepancies that still exist after discussion. For each study, the following data were extracted into data-collection form: the first author's name, publication year, characteristics of participants, trial design, country of study, whole grain diet duration and doses, washout period (only for randomized cross-over trials) and the results of inflammatory markers.

The quality of eligible studies was independently judged by 2 researchers through assessing the risk of bias according to Jadad Scores (7-points) special for randomized controlled trial. Four sources of bias were evaluated: randomization methods, concealment of allocation, double blinding methods, and withdrawals and dropouts.

### 2.3 Statistical analysis

We examined the relation between whole grain diet and inflammatory markers on the basis of the effect estimates and their 95% confidence intervals (CI) in each included report. The amount of whole grain diet and inflammatory marker, such as IL-6, CRP, TNF-α, were measured as Mean with Standard Deviation (SD) that could calculate a standardized mean difference (SMD) with 95% CI. Within each clinical trial, the analysis population was defined as all randomly assigned participants. For the pooled analysis, we used either fixed effect model when heterogeneity was low or random effect model which take heterogeneity within and between studies into account, to calculate the summary SMD. All *P* values were derived from 2-sided tests, and we considered a *P* value less than 0.05 to be statistically significant.

Statistical heterogeneity across studies was assessed by the Cochrane *Q* test and *I*^*2*^ statistics. An *I*^*2*^ value of greater than 50%, or a *P* value less than .05 for the *Q* statistic, was taken to indicate heterogeneity. Subgroup analyses were performed to evaluate the consistency of the whole grain effect within specific subgroups of participants according to their baseline characteristics.

The presence of publication bias was investigated by using the Begg and Egger tests, and results were considered to indicate potential bias at *P* < .05. We also carried out a traditional sensitivity analysis by excluding a study at a time to explore whether the results were driven by a study with extreme result. All data analysis was conducted by STATA software (version 12.0; StataSE). All statistical tests were 2-sided.

## 3 Results

### 3.1 Study selection and study characteristics

**Figure 1:**
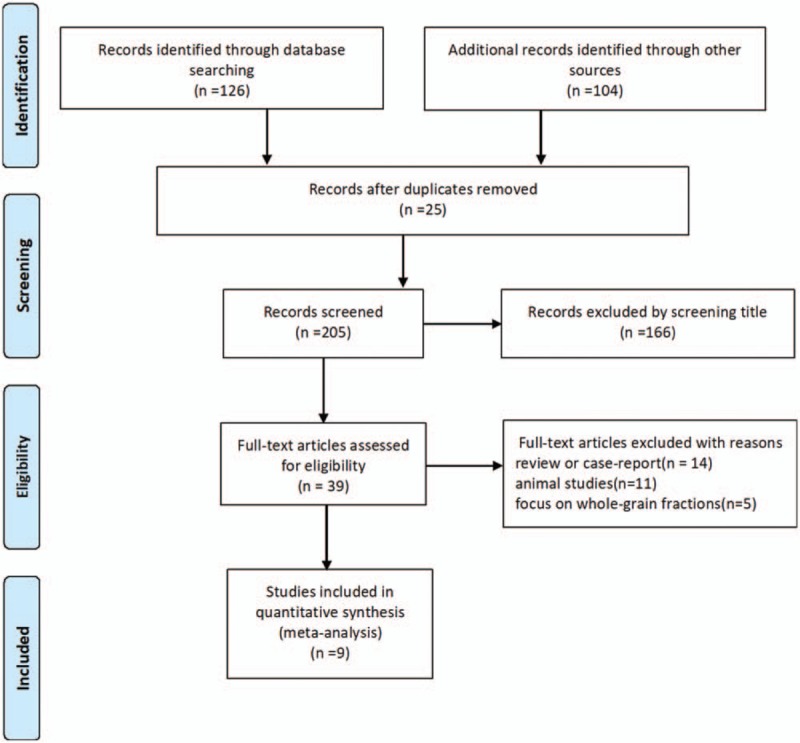
Flow chart of article selection.

The PRISMA flowchart was shown in Figure 1. Initial queries identified a total of 205 articles from all databases and search methods after exclusion of 25 duplicated studies. Screening titles and abstracts yielded 39 full-text reports remaining, among these, 14 review or case-report and 11 animal trials were ruled out, and another 5 studies were not included because they focused on whole-grain fractions, leading to a total of 9 randomized trials finally included in this review. A manual search of the reference lists within the retrieved articles did not yield any new eligible study.

A summary of study characteristics of individual study was presented in Table 1. The included studies enrolled 838 participants randomly assigned to receive either standard diet or whole grain diet, published from 2007 to 2017. Of the 9 studies, 5^[33–37]^ were randomized controlled trial, 4^[32,38–40]^ were randomized crossover study. Among these, 2 studies were conducted in America, 2 in United Kingdom, and each 1 in Australia, Denmark, Iran, Italy, and Sweden, covering different populations and regions.

**Table 1:**
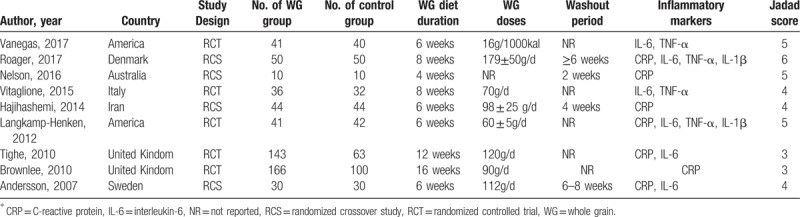
Characteristics of studies investigating associations between whole grain diet and inflammatory markers^∗^.

### 3.2 Quality of the included studies

All 9 studies included in the analysis showed a low risk of selection bias. Randomization and concealment of allocation were performed in 7 studies (77.8%), a double blinding protocol was shown in 6 studies (66.7%), and withdrawals and dropouts details were identified in all studies (100%).

### 3.3 Main analysis

**Figure 2:**
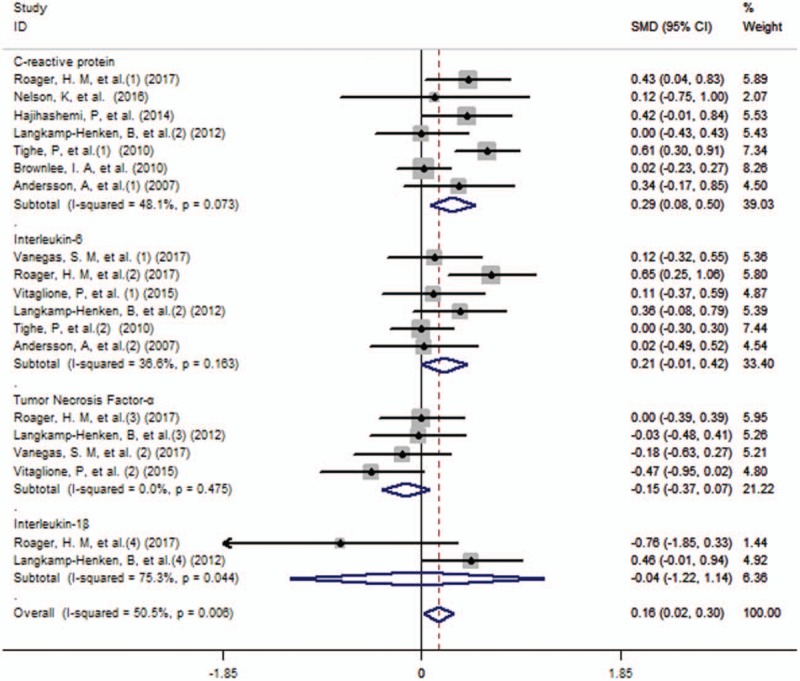
Forest plot (fixed effects model) of whole grain consumption and inflammatory markers, including C-reactive protein, Tumor necrosis factor-α, Interleukin-6 and Interleukin-1β.

For the analyses of the relation of whole grain diet with inflammatory markers: CRP, IL-6, TNF-α, Interleukin-1β (IL-1β), we included 9 randomized trials that enrolled 838 participants reporting data on the issue. In the pooled analysis of all studies, whole grain exposure was significantly associated with the decreased concentration of inflammatory markers (SMD 0.16, 95% CI, 0.02–0.30). Specific analyses for CRP, IL-6, TNF-α, and IL-1β were also conducted, and yielded different outcomes. Among the 4 randomized crossover trials and 3 randomized controlled studies reporting data on CRP, we found that participants with whole grain diet were related with a significant decrease in the concentration of CRP (SMD 0.29, 95% CI, 0.08–0.50) with a low substantial heterogeneity between studies (*I*^*2*^ = 48.1%, *P* for heterogeneity = .07). Meanwhile, there was a significant decrease in the concentration of IL-6 for whole grain intake (SMD 0.19, 95%, 0.03–0.36), on the basis of 4 randomized controlled trials and 2 randomized crossover studies, and no statistically significant heterogeneity was observed (*I*^*2*^ = 36.6%, *P* for heterogeneity = .16). However, there were no statistically significant relation between whole grain diet and the level of TNF-α (SMD −0.15, 95% CI, −0.37 to 0.07) or IL-1β (SMD −0.04, 95%CI, −1.22 to 1.14). The results of main analysis and specific analyses was reported in Table 2 and Figure 2.

**Table 2:**
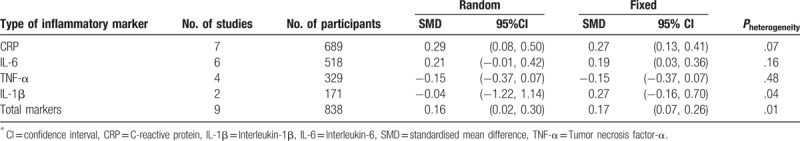
Meta-analysis of whole grain diet and the level of inflammatory markers^∗^.

### 3.4 Subgroup analysis

The summary SMD of CRP and IL-6 with whole grain diet in strata of selected variables were presented in Table 3. As for CRP, subgroup analysis showed that subgroups of “Obese or overweight participants” (SMD 0.35, 95% CI, 0.05–0.66), “>18 years old” (SMD 0.32, 95% CI,0.05–0.59) and “whole grain doses >100 g/day” (SMD 0.51, 95% CI, 0.29–0.72) were associated with significant decrease of CRP, but no relation in the “whole grain diet duration ≥8 weeks”, “whole grain diet duration <8 weeks”, “whole grain doses <100 g/d” subgroups. While no subgroup showed significant result about the concentration of IL-6.

**Table 3:**
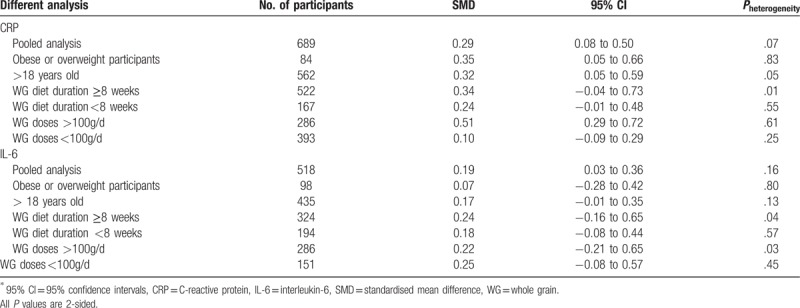
Associations between whole grain diet and inflammatory markers^∗^.

### 3.5 Sensitivity analyses and publication bias

**Figure 3:**
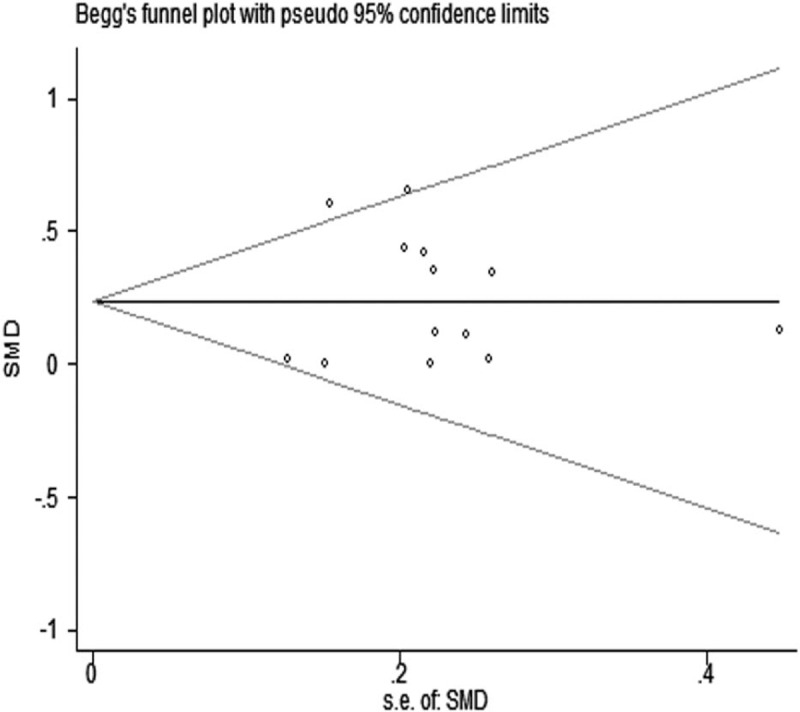
Publication bias of all studies by Begg test.

**Figure 4:**
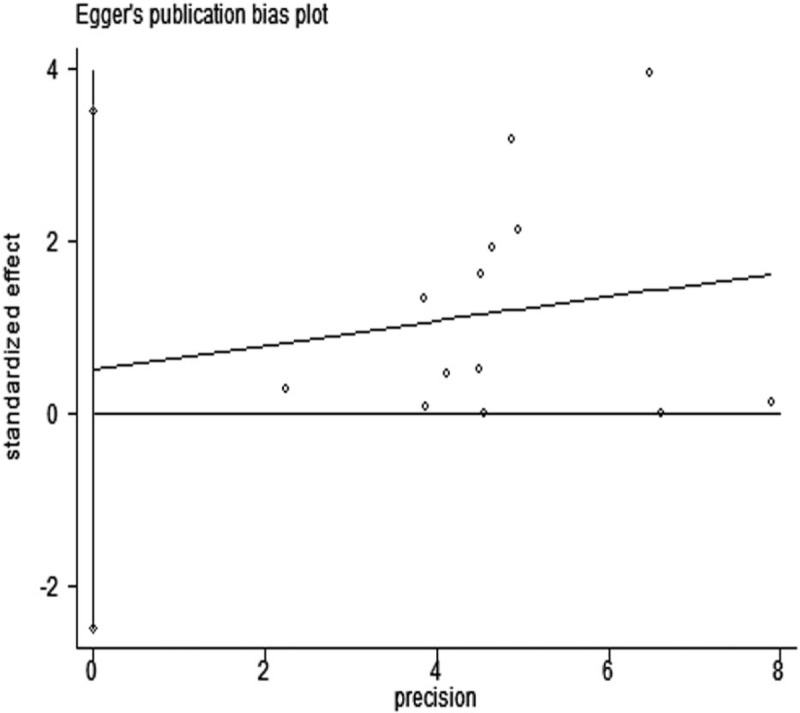
Publication bias of all studies by Egger test.

We also performed sensitivity analyses by withdrawing a study with extreme data and obtained materially unchanged results as in main analysis. Begg and Egger test was conducted to test publication bias between the included studies and did not indicate significant publication bias for Begg test (*P* = .86, Fig. 3) and Egger test (*P* = .71, Fig. 4).

## 4 Discussion

In this pooled analysis of 9 randomized clinical studies with 838 participants from the United states, Australia, Europe, and Asia, we found an overall inverse association between whole grain products intake and inflammatory markers, including CRP, IL-6, TNF-α and IL-1β, matched for overall energy contents and macronutrient compositions. Notably, the inverse association with the concentration of CRP was also found in overweight participants, participants who were more than 18 years old and intake of whole grain that was more than 100 g per day.

The inverse relation between whole grain intake and inflammation had been consistently reported in many observational studies,^[17,41,42]^ but its interpretation remained controversial. First of all, it was reported that gluten and wheat germ agglutinin (WGA) had strong effect in activating pro-inflammatory immune system by increasing intestinal permeability,^[43,44]^ and inducing specific T-cell,^[45]^ but whole grain products contained phytochemicals, like polyphenols, that can exert anti-inflammatory effects by possibly offsetting the effect of gluten and WGA.^[46]^ Secondly, whole grain were known to a source of short-chain fatty acids (SCFA) metabolized by gut microbiota,^[46]^ which had linked to secretion of gut hormones,^[47]^ immune homeostasis,^[48]^ and eventually led to whole grain-duced improvements on low-grade systematic inflammation.^[49]^ Meanwhile, it was proved that SCFA could in particular tight the junction of epithelial cells and increase the transport of secretory IgA to colon, and ultimately preserve the colonic mucosal barrier.^[50–52]^ Finally, a few studies investigated the effect of whole grain on the gut microbiota composition and found a whole grain-induced increase in *Faecalibacterium prausnitzii*, *Prevotella copri*, and Clostridiales.^[53,54]^ Meanwhile, *Erythromicrobium ramosum* was reported to have contributed to the observed reduction in IL-6.^[55]^

In our meta-analysis, the decrease of CRP was also observed in obese participants and adults, that was consistent with previous findings.^[42,56]^ Obesity was associated with increased inflammation and oxidative stress,^[57]^ and whole grain products had been confirmed to have a strong tight with weight loss in adults,^[58]^ so it was possible that whole grain also led to decreased inflammation simultaneously, but this study could not conclude the inverse association in healthy adults owing to the limited trials. Meanwhile, among children and adolescent, consumption of whole grains had been linked to a lower BMI and obesity, but its effect in inflammation was not so clear because only 2 studies retrieved in the meta-analysis.^[35,39]^ Above all, the type of certain whole grain products differed among all the randomized clinical trials, therefore, even though intake of whole grain products more than 100 g per day was observed significant association between decreased inflammation, the recommended concentration of whole grain consumption was needed to treat with caution.

To our knowledge, this was the first meta-analysis conducted to evaluate the association between whole grain products and the concentration of inflammatory markers in randomized clinical trials. For the analysis, we found an inverse relation between them, and the results adjusted for overall energy and other macronutrients. Second, another major strength of our study was the relative larger number of participants, allowing a much greater possibility of performing further subgroup analysis. Third, between-study heterogeneity was small and publication bias was not checked, indicating that our results are stable.

However, there also were some limitations in this meta-analysis. First, the diversity of whole grain as a group of raw materials as well as the food processes used made it difficult to estimate real intake of whole grain, so a biomarker to improve estimation of whole grain was needed for interventional studies with higher quality. Plasma alkylresorcinol had shown great promise as a biomarker due to its stable and absorbable.^[59]^ In addition, several studies had used plasma alkylresorcinol to check compliance, and showed a possible relation between good compliance and outcomes.^[60–62]^ But in this meta-analysis, only 2 studies checked the plasma alkylresorcinol which could exaggerate or diminish the inverse association between whole grain and inflammation.^[32,33]^ Second, of these circulating proteins as markers of inflammation, CRP was most commonly measured, followed by IL-6, and CRP was an acute-phase protein secreted by the liver in response to IL-6.^[63]^ Actually, fibrinogen, IL-2, IL-10, and TNF-α receptor-1 and receptor-2 could also reflect the inflammation status. Yet the meta-analysis was restricted to only the 4 most common markers that may cause the incomplete effect of whole grain on inflammation. Third, heterogeneity was found across studies even it was small, this might have resulted from the differences in design and differences in participants’ characteristics, and most importantly, discrepancy in definition of whole grain products and interventional diet products. Finally, although randomized clinical trials had the greatest ability to exclude confounding factors, the possibility that some other un-measured factors or interaction between nutrients might have been partly responsible for the observed association could not be ruled out.

Furthermore, our meta-analysis pointed out the orientation of prospective studies in this field:

1) Focus on a kind of grain or certain whole grain products to reduce the deviation of different whole grain products;
2) Investigate deeply into the mechanism of the association and the effect of whole grain on health;
3) Enlarge the included participants, such as healthy adults, obese or normal weight children and adolescent;
4) Increase the interventional time of duration, and conduct follow-up to measure the forward effect of whole grain;
5) Add multiple doses of congener whole grain food in 1 intervention to evaluate the effective dosage, ultimately help establish recommended intake in nutrients claim.

In conclusion, higher whole grain intake significantly decreased the concentration of inflammatory markers. From a public health perspective, our findings may provide useful insight and strong evidence in establishing future cereal foods recommendations. However, further well-designed studies are still needed.

## Author contributions

YJX and QYW are co-first authors. JHF, QYW, LD and YJX designed the study. LD, YJX and QYW did the analysis of the data. All the authors contributed to the generation, collection, assembly, interpretation of data. YJX wrote the manuscript under the guidance of YZ and KL. All the authors have read manuscript, and KL and YZ approved the final manuscript.

**Conceptualization:** Jinhua Feng, Liang Du.

**Formal analysis:** Yujie Xu, Qianyi Wan, Liang Du.

**Funding acquisition:** Yong Zhou.

**Methodology:** Qianyi Wan.

**Software:** Jinhua Feng.

**Supervision:** Ka Li, Yong Zhou.

**Validation:** Ka Li.

**Writing – original draft:** Yujie Xu.

## References

[1] Egger G In search of a germ theory equivalent for chronic disease. Prev Chronic Dis 2012;9:E95–101. 22575080 10.5888/pcd9.110301 PMC3431950

[2] Suzuki T Yoshida S Hara H Physiological concentrations of short-chain fatty acids immediately suppress colonic epithelial permeability. Br J Nutr 2008;100:297–305. 18346306 10.1017/S0007114508888733

[3] Hotamisligil GS Shargill NS Spiegelman BM Adipose expression of tumor necrosis factor-alpha: direct role in obesity-linked insulin resistance. Science 1993;259:87–91. 7678183 10.1126/science.7678183

[4] Nahrendorf M Swirski FK Immunology. Neutrophil-macrophage communication in inflammation and atherosclerosis. Science 2015;349:237–8. 26185231 10.1126/science.aac7801

[5] Ross AB Kristensen M Seal CJ Recommendations for reporting whole-grain intake in observational and intervention studies. Am J Clin Nutr 2015;101:903–7. 25809851 10.3945/ajcn.114.098046

[6] Wellen KE Hotamisligil GS Inflammation, stress, and diabetes. J Clin Invest 2005;115:1111–9. 15864338 10.1172/JCI25102 PMC1087185

[7] Williams KJ Tabas I Atherosclerosis and inflammation. Science 2002;297:521–2. 12143880 10.1126/science.297.5581.521

[8] Mizuno Y Jacob RF Mason RP Inflammation and the development of atherosclerosis. J Atheroscler Thromb 2011;18:351–8. 21427505 10.5551/jat.7591

[9] Fernandez-Real JM Ricart W Insulin resistance and chronic cardiovascular inflammatory syndrome. Endocr Rev 2003;24:278–301. 12788800 10.1210/er.2002-0010

[10] Hotamisligil GS Endoplasmic reticulum stress and the inflammatory basis of metabolic disease. Cell 2010;140:900–17. 20303879 10.1016/j.cell.2010.02.034 PMC2887297

[11] Backhed F 99th Dahlem conference on infection, inflammation and chronic inflammatory disorders: the normal gut microbiota in health and disease. Clinical and experimental immunology 2010;160:80–4. 20415855 10.1111/j.1365-2249.2010.04123.x PMC2841839

[12] Fitzgerald EF Hwang S-A Langguth K Fish consumption and other environmental exposures and their associations with serum PCB concentrations among Mohawk women at Akwesasne. Environ Res 2004;94:160–70. 14757379 10.1016/s0013-9351(03)00133-6

[13] Ghayour-Mobarhan M Sahebkar A Vakili R Investigation of the effect of high dairy diet on body mass index and body fat in overweight and obese children. Indian J Pediatr 2009;76:1145–50. 20012799 10.1007/s12098-009-0231-x

[14] Gomez-Garcia A Hernandez-Salazar E Gonzalez-Ortiz M Effect of oral zinc administration on insulin sensitivity, leptin and androgens in obese males. Rev Med Chil 2006;134:279–84. 16676098 10.4067/s0034-98872006000300002

[15] Cepeda-Lopez AC Osendarp SJ Melse-Boonstra A Sharply higher rates of iron deficiency in obese Mexican women and children are predicted by obesity-related inflammation rather than by differences in dietary iron intake. Am J Clin Nutr 2011;93:975–83. 21411619 10.3945/ajcn.110.005439

[16] Katherine E Raffaele M Miryam C Effects of a Mediterranean-style diet on endothelial dysfunction and markers of vascular inflammation in the metabolic syndrome. JAMA 2004;13:7. 10.1001/jama.292.12.1440 15383514

[17] Lutsey PL Jacobs DR Jr Kori S Whole grain intake and its cross-sectional association with obesity, insulin resistance, inflammation, diabetes and subclinical CVD: The MESA Study. Br J Nutr 2007;98:397–405. 17391554 10.1017/S0007114507700715

[18] Meyer KA Kushi LH Jacobs DR Jr Carbohydrates, dietary fiber, and incident type 2 diabetes in older women. Am J Clin Nutr 2000;71:921–30. 10731498 10.1093/ajcn/71.4.921

[19] Aune D Keum N Giovannucci E Whole grain consumption and risk of cardiovascular disease, cancer, and all cause and cause specific mortality: systematic review and dose-response meta-analysis of prospective studies. BMJ 2016;353:i2716–29. 27301975 10.1136/bmj.i2716 PMC4908315

[20] Chan JM Wang F Holly EA Whole grains and risk of pancreatic cancer in a large population-based case-control study in the San Francisco Bay Area, California. Am J Epidemiol 2007;166:1174–85. 17881383 10.1093/aje/kwm194

[21] Schatzkin A Mouw T Park Y Dietary fiber and whole-grain consumption in relation to colorectal cancer in the NIH-AARP Diet and Health Study. Am J Clin Nutr 2007;85:1353–60. 17490973 10.1093/ajcn/85.5.1353

[22] Tang G Wang D Long J Meta-analysis of the association between whole grain intake and coronary heart disease risk. Am J Cardiol 2015;115:625–9. 25727082 10.1016/j.amjcard.2014.12.015

[23] Liu S Manson JE Stampfer MJ Whole grain consumption and risk of ischemic stroke in women: a prospective study. JAMA 2000;284:1534–40. 11000647 10.1001/jama.284.12.1534

[24] Richardson DP Wholegrain health claims in Europe. Proc Nutr Soc 2003;62:161–9. 12749341 10.1079/pns2002226

[25] Marquart L Wiemer KL Jones JM Whole grains health claims in the USA and other efforts to increase whole-grain consumption. Proc Nutr Soc 2003;62:151–60. 12749340 10.1079/pns2003242

[26] Fung TT Rimm EB Spiegeiman D Association between dietary patterns and plasma biomarkers of obesity and cardiovascular disease risk. Am J Clin Nutr 2001;73 1:61–167. 11124751 10.1093/ajcn/73.1.61

[27] Lopez-Garcia E Schulze MB Fung TT Major dietary patterns are related to plasma concentrations of markers of inflammation and endothelial dysfunction. Am J Clin Nutr 2004;80:1029–35. 15447916 10.1093/ajcn/80.4.1029

[28] Jensen MK Koh-Banerjee P Franz M Whole grains, bran, and germ in relation to homocysteine and markers of glycemic control, lipids, and inflammation 1. Am J Clin Nutr 2006;83:275–83. 16469984 10.1093/ajcn/83.2.275

[29] Sahyoun NR Jacques PF Zhang XL Whole-grain intake inversely is associated with the metabolic syndrome and mortality in older adults. Am J Clin Nutr 2006;83:124–32. 16400060 10.1093/ajcn/83.1.124

[30] de Punder K Pruimboom L The dietary intake of wheat and other cereal grains and their role in inflammation. Nutrients 2013;5:771–87. 23482055 10.3390/nu5030771 PMC3705319

[31] Lefevre M Jonnalagadda S Effect of whole grains on markers of subclinical inflammation. Nutr Rev 2012;70:387–96. 22747841 10.1111/j.1753-4887.2012.00487.x

[32] Roager HM Vogt JK Kristensen M Whole grain-rich diet reduces body weight and systemic low-grade inflammation without inducing major changes of the gut microbiome: a randomised cross-over trial. Gut 2017;0:1–1. 10.1136/gutjnl-2017-314786 PMC6839833 29097438

[33] Vanegas SM Meydani M Barnett JB Substituting whole grains for refined grains in a 6-wk randomized trial has a modest effect on gut microbiota and immune and inflammatory markers of healthy adults. Am J Clin Nutr 2017;105:635–50. 28179226 10.3945/ajcn.116.146928 PMC5320415

[34] Vitaglione P Mennella I Ferracane R Whole-grain wheat consumption reduces inflammation in a randomized controlled trial on overweight and obese subjects with unhealthy dietary and lifestyle behaviors: role of polyphenols bound to cereal dietary fiber. Am J Clin Nutr 2015;101:251–61. 25646321 10.3945/ajcn.114.088120

[35] Langkamp-Henken B Nieves C Jr Culpepper T Fecal lactic acid bacteria increased in adolescents randomized to whole-grain but not refined-grain foods, whereas inflammatory cytokine production decreased equally with both interventions. J Nutr 2012;142:2025–32. 23014489 10.3945/jn.112.164996

[36] Tighe P Duthie G Vaughan N Effect of increased consumption of whole-grain foods on blood pressure and other cardiovascular risk markers in healthy middle-aged persons: a randomized controlled trial. Am J Clin Nutr 2010;92:733–40. 20685951 10.3945/ajcn.2010.29417

[37] Brownlee IA Moore C Chatfield M Markers of cardiovascular risk are not changed by increased whole-grain intake: the WHOLEheart study, a randomised, controlled dietary intervention. Br J Nutr 2010;104:125–34. 20307353 10.1017/S0007114510000644 PMC3501710

[38] Nelson K Mathai ML Ashton JF Effects of malted and non-malted whole-grain wheat on metabolic and inflammatory biomarkers in overweight/obese adults: a randomised crossover pilot study. Food Chem 2016;194:495–502. 26471584 10.1016/j.foodchem.2015.08.023

[39] Hajihashemi P Azadbakht L Hashemipor M Whole-grain intake favorably affects markers of systemic inflammation in obese children: a randomized controlled crossover clinical trial. Mol Nutr Food Res 2014;58:1301–8. 24478050 10.1002/mnfr.201300582

[40] Andersson A Tengblad S Karlstom B Whole-grain foods do not affect insulin sensitivity or markers of lipid peroxidation and inflammation in healthy, moderately overweight subjects. J Nutr 2007;137 6:1401–7. 17513398 10.1093/jn/137.6.1401

[41] Gaskins AJ Mumford SL Rovner AJ Whole grains are associated with serum concentrations of high sensitivity C-reactive protein among premenopausal women. J Nutr 2010;140:1669–76. 20668255 10.3945/jn.110.124164 PMC2924598

[42] Masters RC Liese AD Haffner SM Whole and refined grain intakes are related to inflammatory protein concentrations in human plasma. J Nutr 2010;140:587–94. 20089789 10.3945/jn.109.116640 PMC2821887

[43] Maes M Mihaylova I Leunis JC Increased serum IgA and IgM against LPS of enterobacteria in chronic fatigue syndrome (CFS): indication for the involvement of gram-negative enterobacteria in the etiology of CFS and for the presence of an increased gut-intestinal permeability. J Affect Disord 2007;99:237–40. 17007934 10.1016/j.jad.2006.08.021

[44] Dalla Pellegrina C Perbellini O Scupoli MT Effects of wheat germ agglutinin on human gastrointestinal epithelium: insights from an experimental model of immune/epithelial cell interaction. Toxicol Appl Pharmacol 2009;237:146–53. 19332085 10.1016/j.taap.2009.03.012

[45] Troncone R Jabri B Coeliac disease and gluten sensitivity. J Intern Med 2011;269:582–90. 21481018 10.1111/j.1365-2796.2011.02385.x

[46] Fardet A New hypotheses for the health-protective mechanisms of whole-grain cereals: what is beyond fibre. Nutr Res Rev 2010;23:65–134. 20565994 10.1017/S0954422410000041

[47] Wichmann A Allahyar A Greiner TU Microbial modulation of energy availability in the colon regulates intestinal transit. Cell Host Microbe 2013;14:582–90. 24237703 10.1016/j.chom.2013.09.012

[48] Furusawa Y Obata Y Fukuda S Commensal microbe-derived butyrate induces the differentiation of colonic regulatory T cells. Nature 2013;504:446–50. 24226770 10.1038/nature12721

[49] Martinez I Lattimer JM Hubach KL Gut microbiome composition is linked to whole grain-induced immunological improvements. ISME J 2013;7:269–80. 23038174 10.1038/ismej.2012.104 PMC3554403

[50] Cani PD Possemiers S Van de Wiele T Changes in gut microbiota control inflammation in obese mice through a mechanism involving GLP-2-driven improvement of gut permeability. Gut 2009;58:1091–103. 19240062 10.1136/gut.2008.165886 PMC2702831

[51] Kvale D Brandtzaeg P Butyrate differentially affects constitutive and cytokine-induced expression of HLA molecules, secretory component (SC), and ICAM-1 in a colonic epithelial cell line (HT-29, clone m3). Adv Exp Med Biol 1995;371A:183–8. 8525902 10.1007/978-1-4615-1941-6_37

[52] Commane DM Shortt CT Silvi S Effects of fermentation products of pro- and prebiotics on trans-epithelial electrical resistance in an in vitro model of the colon. Nutr Cancer 2005;51:102–9. 15749636 10.1207/s15327914nc5101_14

[53] Ramirez-Farias C Slezak K Fuller Z Effect of inulin on the human gut microbiota: stimulation of Bifidobacterium adolescentis and Faecalibacterium prausnitzii. Br J Nutr 2009;101:541–50. 18590586 10.1017/S0007114508019880

[54] Kovatcheva-Datchary P Nilsson A Akrami R Dietary fiber-induced improvement in glucose metabolism is associated with increased abundance of prevotella. Cell Metabol 2015;22:971–82. 10.1016/j.cmet.2015.10.001 26552345

[55] Le Chatelier E Nielsen T Qin J Richness of human gut microbiome correlates with metabolic markers. Nature 2013;500:541–6. 23985870 10.1038/nature12506

[56] Van Dam QIL Liu RM Franz S Whole-grain, bran, and cereal fiber intakes and markers of systemic inflammation in diabetic women. Diabetes Care 2006;29 2:207–11. 16443861 10.2337/diacare.29.02.06.dc05-1903

[57] Lakshman R Elks CE Ong KK Childhood obesity. Circulation 2012;126:1770–9. 23027812 10.1161/CIRCULATIONAHA.111.047738 PMC3785130

[58] Pol K Christensen R Bartels EM Whole grain and body weight changes in apparently healthy adults: a systematic review and meta-analysis of randomized controlled studies. Am J Clin Nutr 2013;98:872–84. 23945718 10.3945/ajcn.113.064659

[59] Ross AB Present status and perspectives on the use of alkylresorcinols as biomarkers of wholegrain wheat and rye intake. J Nutr Metabol 2012;462967:1–2. 10.1155/2012/462967 PMC3270436 22363838

[60] Ross AB Bourgeois A Macharia HN Plasma alkylresorcinols as a biomarker of whole-grain food consumption in a large population: results from the wholeheart intervention study. Am J Clin Nutr 2012;95:204–11. 22170369 10.3945/ajcn.110.008508 PMC3592483

[61] Kristensen M Toubro S Jensen MG Whole grain compared with refined wheat decreases the percentage of body fat following a 12-week, energy-restricted dietary intervention in postmenopausal women. J Nutr 2012;142:710–6. 22357746 10.3945/jn.111.142315

[62] Ampatzoglou A Atwal KK Maidens CM Increased whole grain consumption does not affect blood biochemistry, body composition, or gut microbiology in healthy, low-habitual whole grain consumers. J Nutr 2015;145:215–21. 25644340 10.3945/jn.114.202176

[63] Rifai N Ridker PM Inflammatory markers and coronary heart disease. Curr Opin Lipidol 2002;13:383–9. 12151853 10.1097/00041433-200208000-00005

